# Psychedelic ego-dissolution: a philosophical and qualitative critique

**DOI:** 10.1098/rstb.2025.0154

**Published:** 2026-11-10

**Authors:** Jason K. Day, Dan Zahavi

**Affiliations:** ^1^ Molecular Psychiatry Lab, Faculty of Science and Medicine, University of Fribourg, Fribourg, Switzerland; ^2^ Center for Subjectivity Research, University of Copenhagen, Copenhagen, Denmark

**Keywords:** ego-dissolution, psychedelics, 5-MeO-DMT, self-consciousness, subjectivity, for-me-ness

## Abstract

Ego-dissolution is a phenomenon of central interest across interdisciplinary research on psychedelics. In this paper, we present a philosophical and qualitative critique of the claim that ego-dissolution can result in conscious states with no phenomenal sense of self, i.e., states that completely lack any form of self-consciousness. We first turn to recent publications by Chris Letheby and Raphaël Millière, for whom trip reports of experiences induced by 5-MeO-DMT (5-methoxy-N,N-dimethyltryptamine) present clear evidence for the existence of totally selfless experiential states. Their claim is undermined, we argue, by the fact that they operate with overinflated notions of ‘self’ and ‘self-consciousness,’ meaning that more minimal forms are available and arguably still present in 5-MeO-DMT experiences. We then perform a qualitative thematic analysis of 411 5-MeO-DMT trip reports. Pointing to the ambiguous language of the trip reports, the self-admitted inadequacy of the descriptions and the affective dimension of the reported experiences, we argue that claims concerning total selflessness are unwarranted and instead offer an alternative interpretation: extreme ego-dissolution during 5-MeO-DMT experiences involves a radically minimal form of self-consciousness, one in which the identity of the subject is experienced as unfamiliar and underdetermined.

This article is part of the discussion meeting issue ‘From being to identity: analysis and synthesis of the self’.

## 1. Introduction

The past decade has seen an abundance of publications discussing ‘ego- dissolution’ induced by the intake of psychedelic substances (LSD (lysergic acid diethylamide), N,N-DMT (dimethyltryptamine), psilocybin, mescaline, and 5-MeO-DMT (5-methoxy-N,N-dimethyltryptamine)), including its psychotherapeutic benefits, neural correlates and what it might tell us about the fundamental nature of consciousness (e.g. [1–4]). Indeed, ego-dissolution is among the most popular notions in contemporary psychedelic research and has even been identified as a defining characteristic of psychedelic experiences (e.g. [5–7]). In this paper, we will not only voice concerns about some of the claims repeatedly found in the literature on ego-dissolution but also offer an analysis of our own based on an extensive analysis of first-person trip reports. We are especially concerned here with claims made by Chris Letheby [8] and Raphaël Millière [9] according to which 5-MeO-DMT trip reports present clear evidence that ego-dissolution can lead to totally selfless states of consciousness.

Before starting out, however, it is necessary to make a conceptual clarification. A variety of different terms, including ‘ego disintegration,’ ‘ego loss,’ ‘ego death’ and ‘ego-dissolution’, have been employed as descriptors for experiences typically taken to involve a ‘(reversible) loss of one's sense of self and sense of self-world boundaries’ [9, p.19]. This phrasing not only suggests that the terms ‘ego’ and ‘self’ are frequently used interchangeably, but also points to the vexed relation between metaphysics and experience. Although some authors have argued that experiences of self-loss ultimately reveal the non-existence of the self, i.e., its illusory nature, others have been more cautious and insisted that we ought to distinguish the metaphysical question of whether an experience involves an experiencer from the question of whether the self necessarily figures in experience [9, p.27]. In the following, we will also adopt the latter approach and therefore frame and phrase our discussion of ‘ego-dissolution’ as a discussion of self-consciousness. In short, when asking whether the intake of 5-MeO-DMT can induce states of total selflessness, the question is not whether 5-MeO-DMT can literally dissolve or disintegrate the metaphysical bearer or owner of the experiences, but rather whether it can induce states without any phenomenal ‘sense of self,’ i.e., states that, to quote Millière, completely lack ‘self-consciousness’ [9, p.27].

## 2. Ego-dissolution according to Letheby and Millière

Much current research on ego-dissolution has focused upon studying its psychotherapeutic relevance for mental health and well-being (see [3] for a systematic review of these studies). Ego-dissolution has, for instance, been found to be significantly associated with reductions in symptoms of depression, stress, anxiety and substance use disorders (e.g. [10–14]). But one decisive problem with these studies is their conceptual vagueness (cf. [15]). They feature, if at all, single-sentence definitions of ego-dissolution. For example:

— a dissolution of the ordinary sense of self and a breakdown of the perceived boundaries between the self and the world [16, p.1].
— an experience of a sense of unity with the world and other people, and disintegration of a coherent concept of ‘self’ [17, p.988].

In both cases, the notion of self is left undefined, as are the attendant concepts of ‘dissolution,’ ‘disintegration’ and ‘unity’.

We encounter the same type of vagueness in the psychometric instruments predominantly used to assess participant experience. Consider, for instance, the *Ego-Dissolution Inventory* (EDI) [6]. The EDI is a self-report rating scale for the purposes of measuring participants’ experience of ego-dissolution. It is comprised of eight items to which participants respond by rating their experience on a scale of 0 (‘no, not more than usual’) to 100 (‘yes, entirely/completely’). Some examples are: ‘I experienced a dissolution of my ‘self’ or ego,’ ‘I experienced a disintegration of my ‘self’ or ego,’ ‘I lost all sense of ego’ and ‘all notion of self and identity dissolved away’ [6, p.3]. But none of the items provides any specification to the participants as to the meaning of ‘self’ or ‘ego’ employed, as well as the meaning of the inquired ‘disintegration,’ ‘dissolution’ or ‘loss’ thereof. Of course, the degree to which psychometric items can provide conceptual detail is limited by pragmatic requirements concerning readability for participants. Nonetheless, improved conceptual clarity of key terms is desirable, at least for researchers. Recent philosophical work has tried to remedy this situation by offering explicit definitions of the terms in question, but as we shall see, these accounts have problems of their own.

One of the first philosophical engagements with the topic of ego-dissolution was offered by Letheby & Gerrans [18], who claimed that ego-dissolution should be understood as ‘a phenomenon of unbinding’ [18, p.2], i.e., as a disintegration of our usual unified and coherent self-representation. More specifically, Letheby & Gerrans argued that ego-dissolution during psychedelic experiences offers clear evidence against the claim that consciousness is always imbued with a sense of self and always characterized by self-consciousness.^1^ For both, self-consciousness is to be understood as the experience of being a prime mover, a persisting unified entity that is distinct from the experiences, thoughts and feelings that it owns and sustains [18, p.2]. To be self-conscious, in short, is to have the experience of being a single unified immaterial substance [18, p.2]. Letheby & Gerrans deny the existence of such an entity and consequently take the phenomenon of ego-dissolution to involve the dissipation of a misrepresentation, and therefore to contain an element of veracity; it shows what there really is, namely, no self [18, p.1].

In a subsequent sole-authored publication, Letheby [8] offers a more fine-grained defence of the view that there are experiences that completely lack self-consciousness, but now operates with a different conception of self-consciousness. He frames his discussion in terms of two sets of distinctions originally introduced by Farell & McClelland [19] and Guillot [20], respectively: the distinction between *universalism*, *typicalism* and *absentism*, on the one hand, and *for-me-ness*, *mineness* and *me-ness* on the other. To start with the latter trio, for-me-ness, for Guillot, is a label for the special inner awareness that the subject necessarily has of the experience she is undergoing. Importantly, insofar as for-me-ness consists in a subject's awareness of their mental states, it constitutes an implicit form of self-consciousness [8, p.13].^2^ Me-ness refers to the situation where the subject of experience, rather than simply being aware of the external object (and of the experience of the object), is also aware of herself. Mineness, finally, designates the situation where the experience is phenomenally given *as* mine. On this reading, mineness is the more complex notion, since it not only requires that the subject be aware of her experience, and aware of herself, but also aware of the possessive relation between herself and the experience—that is, aware that she is the owner of the experience [20, pp.27–32]. As for the distinction between universalism, typicalism and absentism, these three notions are taken by Letheby to in turn designate the claim that all experiences are characterized by for-me-ness, that ordinary experiences are characterized by for-me-ness, and that no experiences are characterized by for-me-ness. Ultimately, Letheby defends typicalism, since he on the one hand takes the vast majority of human conscious experiences to feature for-me-ness [8, pp.22–23], but on the other also argues that there are psychedelic states—most notably those induced by 5-MeO-DMT—that lack for-me-ness and any form of self-consciousness altogether [8, p.17]. A description of allegedly total ego-dissolution that Letheby keeps referring to as evidence for this claim is Pollan's account of what happened when he took 5-MeO-DMT:

…terror seized me—and then, like one of those flimsy wooden houses erected on Bikini Atoll to be blown up in the nuclear tests, ‘I’ was no more, blasted to a confetti cloud by an explosive force I could no longer locate in my head, because it had exploded that too, expanding to become all that there was. Whatever this was, it was not a hallucination. A hallucination implies a reality and a point of reference and an entity to have it. None of those things remained. Unfortunately, the terror didn't disappear with the extinction of my ‘I’. Whatever allowed me to register this experience, the post-egoic awareness I'd first experienced on mushrooms, was now consumed in the flames of terror too. In fact, every touchstone that tells us ‘I exist’ was annihilated, and yet I remained conscious. ‘Is this what death feels like? Could this be it?’ That was the thought, though there was no longer a thinker to have it [21, pp.276–277].

As Letheby puts it, given descriptions of 5-MeO-DMT experiences such as Pollan's, one must recognize the existence of episodes of selfless phenomenality, i.e., experiences that lack self-consciousness altogether, experiences that do not even retain for-me-ness [8, pp. 15, 17].

Currently, the most conceptually sophisticated account of ego-dissolution can be found in the work of Millière. In a number of articles, Millière has not only argued that there are different aspects of selfhood and different forms of self-consciousness, but also that there is no such thing as ‘ego-dissolution’ in absolute terms. Rather, it is an umbrella term, and instead of using it indiscriminately, one should realize that it can take different forms and that it can occur to different degrees. Accordingly, psychedelics can disrupt different aspects or components of self-consciousness [9, p.21, 22, p.2]. In one of his articles, Millière argues that one can find six main notions of self-consciousness in the literature: (i) *cognitive self-consciousness*, i.e., thinking of oneself as oneself; (ii) *spatial self-consciousness*, i.e., being conscious of one's location as one's own; (iii) *sense of bodily ownership*, i.e., being conscious of one's body as one's own; (iv) *sense of bodily agency*, i.e., being conscious of one's bodily actions as one's own; (v) *sense of mental ownership*, i.e., being conscious of one's mental states as one's own, and (vi) *sense of mental agency*, i.e., being conscious of one's mental actions as one's own [9, p.5]. Each of these notions might be either present or absent in different types of experiences, but as Millière then also argues, some psychedelic-induced experiences, in particular experiences induced by 5-MeO-DMT, can plausibly lack all six forms of self-consciousness [9, p.20], and consequently qualify as truly selfless states [9, p.6]. Millière likewise distinguishes a high-level narrative sense of self (that involves self-referential thought and the capacity for mental time travel) from a more minimal embodied sense of self (that includes body ownership, body awareness and spatial self-location) and argues that both can be disrupted or entirely lost in psychedelic experiences [9, p.29, 23, p.11]. As part of the available evidence, Millière offers the same quote by Pollan as Letheby [9], but also several reports from the Erowid.org database, including the following 5-MeO-DMT report: ‘there was no self, nothing to separate me from the sensation’ ([24] quoted in [9, pp.22–23]).

## 3. Philosophical critique

To suggest, as Letheby and Gerrans do, that the self-consciousness we ordinarily enjoy is the experience of being a Cartesian ‘substance, a prime mover, a persistent entity that underlies, owns and initiates thoughts, experiences and actions’ [18, p.8], and that this Cartesian intuition furthermore remains prevalent ‘in philosophical discussions of selfhood’ [18, p.9] is a surprising claim. Anybody familiar with theoretical discussions of self and self-consciousness within philosophy, psychology, sociology or the cognitive sciences would know that this is not the received view. For a simple illustration, consider Ulric Neisser's influential distinction between five different types of self-knowledge and five senses of self (the *ecological*, *interpersonal*, *extended*, *private* and *conceptual*), which, although ‘rarely experienced as distinct […] differ in their developmental histories, in the accuracy with which we can know them, in the pathologies to which they are subject’ [25, p.35]. Generally speaking, most of the prominent theoretical work on self and self-consciousness in the twentieth and twenty-first centuries, including work by figures such as Husserl, Mead, Heidegger, Merleau-Ponty, Sartre, Henrich, Frank, Castañeda, Shoemaker, Taylor, Kriegel, Schechtman, Strawson, Zahavi, etc., bears no resemblance to the definitions provided by Letheby and Gerrans. In fact, it is perhaps not entirely coincidental that two other thinkers who happen to endorse the same definition of self as Letheby and Gerrans—namely, Metzinger [26] and Garfield [27]—are both seeking to establish the non-existence of self. To put it differently, the definition in question is primarily endorsed by people who want to deny the reality of selfhood.

Just like Letheby, Millière also argues that ego-dissolution is philosophically intriguing since it provides clear empirical evidence against the claim that (phenomenal) consciousness is necessarily characterized by self-consciousness [23, p.13, 28, p.897]. But the validity of this claim depends on what is meant by self-consciousness. We have seen that Millière distinguished six different notions and that he concluded that all were absent in certain psychedelic states. However, there are more notions to consider. When talking of self-consciousness, the notion can be given both an egological and a non-egological reading [29]. The latter distinction, which can already be found in classical discussions of self-consciousness (e.g. [30]), bears on the question of whether self-consciousness is best understood as consciousness of self or rather as the acquaintance that consciousness has with itself. When considering authors who have defended the view that consciousness is necessarily characterized by self-consciousness, it is noteworthy that they have typically been operating with the second, more minimalist and reflexivist conception. Here are a few representative quotes.

— What would it be like to be conscious of something without being aware of this consciousness? It would mean having an experience with no awareness whatever of its occurrence. This would be, precisely, a case of unconscious experience. It appears, then, that being conscious is identical with being self-conscious. Consciousness is self-consciousness [31, p.162].
— It is impossible to think or experience something consciously without thinking or experiencing it self-consciously, i.e., without being peripherally aware of thinking or experiencing it [32, p.200].
— Just as an illuminating light is considered to be the illuminator of itself, because of its nature, just so, awareness is aware of itself (Dharmakīrti quoted in [33, p.53]).

Where does Millière stand on this? In a recent article on the topic, he endorses the view that ‘a mental state is a conscious mental state if and only if its subject is pre-reflectively conscious of it—that is, if and only if it has ‘for-me-ness’’ [34, p.627]. In short, he accepts the claim that consciousness is necessarily characterized by a minimal pre-reflective self-consciousness, just as he accepts universalism regarding for-me-ness. He consequently defends a position that is markedly different from Letheby's. In an earlier publication, however, Millière explicitly criticizes the necessity claim, i.e., the view that self-consciousness is necessary for consciousness in general, and instead endorses the typicality claim: the view that self-consciousness is merely pervasive in ordinary experience [9, pp.2, 34]. At the same time, he also argues that even if psychedelic experiences involving ego-dissolution might not be bereft of for-me-ness, they are still totally selfless [9, p.29]. How to resolve this tension? The first part of the explanation is relatively simple and ultimately terminological—a point that Millière explicitly acknowledges. In his view, it is in the end best to reserve the notion of self-consciousness for egological accounts only. The fact that the notion is also being used as a label for the self-reflexive or self-intimating character of consciousness is merely generating confusion and misunderstanding [9, p.29, 34, p.627]. There is an obvious problem with this terminological stipulation, however.^3^ The latter use of the term is not only perfectly legitimate and lexically justified but also widespread, not only in twentieth century philosophy (Husserl, Sartre, Gurwitsch, Henrich, Frank and Kriegel to mention just a few), but also more generally in the history of philosophy, be it Western or Indian, and it seems rather odd to mandate against its use on the basis of some recent misunderstandings, many of which can be traced back to a single special issue of *Review of Philosophy and Psychology* [36].^4^

A more cogent alternative would be to describe psychedelic experiences as involving alterations or transformations of self-consciousness and to concede that although a variety of subtypes of self-consciousness might be completely lost, there is also one fundamental type that remains, namely, the one that is constitutive of phenomenal consciousness.

This reasoning, however, still leaves one important question unanswered. If the self-consciousness that remains in place is aptly characterized in terms of for-me-ness, can it then still be totally selfless? The answer depends on what one means by ‘self.’ For Millière, the most minimal notion of self is the embodied notion, i.e., the one that includes a sense of body ownership, of body awareness and, most fundamentally, of spatial self-location [22, p.8, 23, pp. 10–11]. It is, however, unclear why Millière makes this claim, since the current literature contains discussions of more minimalist conceptions of self (e.g. [38,39]). Rather than conceiving of the self as some*thing* that has experiences or arguing that the most basic sense of self involves spatial and bodily awareness, it is also possible to define what has been called the *minimal self* in processual and experiential terms as the very first-personal character of experience, i.e., as the very subjectivity of experience. On this account, experiences are always given perspectivally. They come with a subjective point of view, a for-*me*-ness, insofar as they, when lived through, are given in a manner that is not available to others. Experiences possess for-me-ness in virtue of their self-intimating or self-presentational nature. For-me-ness is consequently not to be understood as some extra experiential ingredient, some distinct self-quale, nor is it something that depends upon or requires a capacity for reflection or articulation [37,40,41]. Presumably Millière would accept this characterization of phenomenal consciousness but dispute that it is sufficient for selfhood. In the end, it consequently appears that a significant part of the discussion between no-selfers and pro-selfers is verbal rather than substantial and centred around the question of how deflationary or thin a notion of self one operates with.

We can illustrate this point by way of a small digression into Buddhist philosophy. In a recent paper, MacKenzie discussed the relation between phenomenal consciousness, minimal subjectivity and selfhood [33]. MacKenzie is a scholar of Buddhist philosophy and a defender of a view already to be found in Dignāga (480–540 CE) and Dharmakīrti (600–670 CE) known as dual-aspect reflexivism or self-illuminationism. According to the view in question, phenomenal consciousness involves something it is like to have an experience and that in turn ‘is a function of how the experience presents the object and how it presents itself’ [33, p.51]. For MacKenzie, just like for Frankfurt, phenomenal consciousness is necessarily characterized by a form of reflexive self-givenness or self-consciousness that MacKenzie also terms minimal subjectivity. In a subsequent move, MacKenzie then insists that this minimal subjectivity must be distinguished from selfhood. The former is necessary but not sufficient for the latter. How does he define selfhood? MacKenzie argues that selfhood involves at least four capacities or features, namely (i) subjectivity, (ii) ownership, (iii) agency and (iv) valuation [33]. Accordingly, although there cannot be experiences without subjectivity, there can be experiences without a sense of self. But consider again how MacKenzie speaks of subjectivity. He claims that subjectivity involves a self/other contrast and that it amounts to a basic form of self-acquaintance that in turn constitutes the most basic form of a first-person perspective [33, pp.40, 58–59]. And at this point, the obvious question to ask is why the presence of self-acquaintance, a lived first-person perspective and a self–other contrast is not enough to justify operating with a minimal sense of self?

In the end, what ultimately matters is not the self-label, but the question of whether our experiential life is as such characterized by self-consciousness, subjectivity and for-me-ness. If the latter is indeed the case, it seems recommendable to opt for the least misleading terminology, since this will help to keep the discussion substantial. In our view, choosing to designate experiences that possess self-consciousness, subjectivity and for-me-ness as ‘selfless’ would be the wrong choice. It is one thing, however, to disagree about the choice of label as Millière does. It is another thing to question the characterization, which is something that Letheby has done.

As Shoemaker once wrote, it is ‘essential to a philosophical understanding of the mental that we appreciate that there is a first-person perspective on it, a distinctive way mental states present themselves to the subjects whose states they are’ [42, p.157]. Letheby would disagree. He does not deny that, say, Pollan is better placed than anybody else to report on his own 5-MeO-DMT experience, but he does deny that the special epistemic access that subjects have to their own experiences is in any way owing to those experiences being *given* in a distinct manner to them. As Letheby puts it, we should not conflate uncontroversial claims concerning epistemic asymmetry with questionable claims about phenomenal features [8, p.20].

But how should we understand this denial? Is the claim that the experience that a subject is reporting on is not phenomenally given or manifest to that subject at all, or is the claim rather that the experience is phenomenally given but in a manner that is intersubjective and third-personal rather than subjective and first-personal? Both proposals would jeopardize standard accounts of both first-person authority and interpersonal understanding. When I am claiming that I am having a headache, my claim is based on different experiential evidence from my claim that you are having a headache.^5^ In addition, however, it is hard to reconcile Letheby's denial with his own admission that for-me-ness understood as an experiential feature figures in the ‘structure of the vast majority of phenomenal states’ [8, p.17]. Perhaps Letheby would claim that Pollan's 5-MeO-DMT experiences are given to Pollan without the extra phenomenal ingredient that supposedly normally marks our experiences *as* ours. One might wonder whether our ordinary experiences really do possess such a feature; but more importantly, if this is indeed what Letheby is claiming, it would be beside the point because he would then no longer be discussing *for-me-ness* but *mineness* (to use his own and Guillot's terminology).

What remains clear though, is that for Letheby, Pollan's 5-MeO-DMT experience lacks any sense of self. Another description of a 5-MeO-DMT experience quoted by Letheby to illustrate a state that involves a loss of all sense of self is the following:

when I say, ‘I couldn't see’, you shouldn't think that I was seeing blackness as a symbol of nothingness. I truly saw nothing. I ceased to have any memory of that sense […]. Fortunately, despite the extreme sensory deprivation, I was still having a great experience […]. I entered a state of infinite bliss that was so incredible that it seemed totally impossible […]. The joy and ecstasy [*sic*] I felt was absolutely infinite ([48] quoted in [8, p.12]).

But why should this description, with its explicit reference to experiences undergone by the subject, naturally lend itself to such an interpretation? Given the extent to which much of the discussion of whether ego-dissolution can result in totally selfless experiences ultimately rests on conceptual and terminological stipulations, it is clearly worth taking another, more careful look at the trip reports. This is especially needed because their use by Letheby and Millière is methodologically underdetermined. Let us show what a more methodologically rigorous study of these reports can unearth.

## 4. Qualitative critique

Millière [9] and Letheby [8] present first-person self-reports, i.e., ‘trip reports,’ of 5-MeO-DMT experiences as their strongest evidence for the existence of totally selfless conscious states. Before evaluating this evidence, there are some methodological issues with their accounts that must first be addressed.

Both Millière and Letheby base their claims on a rather limited sample of reports and offer no description of the method by which they have selected and analysed these samples. As evidence, Millière presents short extracts from 23 trip reports of psychedelic experiences in total, with 19 of these being 5-MeO-DMT trip reports (the majority being taken from Erowid.org). For his part, Letheby cites short extracts from just four trip reports in total, two of which are 5-MeO-DMT reports and two of which are the same reports that are cited by Millière. There are currently 412 5-MeO-DMT trip reports available on Erowid.org alone—the largest online database of trip reports. Given the available evidence, an analysis of only 21 5-MeO-DMT trip reports, i.e., around 5% of those that are available on Erowid.org, is insufficient for making strong claims regarding how ego-dissolution is ‘typically’ experienced and ‘frequently described’ by subjects who have taken 5-MeO-DMT [8, pp.9, 11]. To be clear, neither Millière, Letheby, nor we ourselves are primarily concerned with the question of exactly how frequently ego-dissolution is experienced on 5-MeO-DMT. The key issues at hand concern what the most plausible characterisation of 5-MeO-DMT-induced ego-dissolution is, and most importantly, whether there is any compelling evidence for it involving total selflessness.

Nonetheless, making strong claims about the nature of ego-dissolution and the existence of total selflessness on the basis of a very limited set of reports runs several risks. The main problem is not that one might be over-generalizing on the basis of a few cherry-picked examples. This problem could after all be alleviated by arguing that all that really matters is whether it is possible to find just one single trip report that unequivocally describes a state of total selflessness, since this would conclusively disprove the view that self-consciousness is ubiquitous to all conscious states. The main problem concerns the ambiguous language of the trip reports at hand. As already pointed out, terms such as ‘self,’ ‘ego,’ ‘I,’ ‘me’ and ‘self-consciousness,’ not to speak of ‘dissolution’ or ‘disintegration,’ are not terms for which people typically have clear and well-worked-out theoretical understanding (cf. [15]). In view of this, one cannot simply take a few trip reports at ‘face value,’ as Letheby [8, pp.8, 10] states one must, nor can one assume that a self-reporter who uses the terms noted above is using them in a way that corresponds to how they are being discussed and defined in the philosophical literature (see also [49]). What's more, we will show in the course of our analysis that the trip reporters themselves profess that their experience of ego-dissolution was of such a bizarre nature that they are unable to accurately describe, comprehend or even remember it. What we wish to demonstrate, with proper recognition of these issues, is that by comparing many different descriptions of ego-dissolution, by considering alternate ways of articulating the experience, one might not only establish more reliable commonalities between the many reported experiences but also obtain a clearer and more multifaceted grasp of the phenomenon in question.

The accounts of Millière and Letheby have been criticized in the past [2,49,50], but none of these earlier criticisms has included more comprehensive qualitative analyses. Let us now rectify this omission.

### (a) Analysis of 5-MeO-DMT trip reports

#### (i) Method

We collected 412 anonymous 5-MeO-DMT trip reports from Erowid.org (an online database of anonymised trip reports made publicly available). This is the complete curated collection of 5-MeO-DMT trip reports in the database as of 23.12.2024.^6^ One hundred of these reports involve the use of 5-MeO-DMT in combination with other psychoactive substances, and in 13 reports there was a potential conflation between N, N-DMT and 5-MeO-DMT. We did not exclude such reports from our analysis since we are not concerned with ‘pure’ 5-MeO-DMT experiences but rather with any experience involving 5-MeO-DMT and ego-dissolution. We excluded three reports for not containing first-person descriptions of the experience [51–53].^7^ In addition to the 409 reports finally included for study from Erowid.org, we included two reports published elsewhere [21, pp.272–290, 54, pp.16–22] as these are cited as examples by Millière and Letheby. A total of 411 trip reports were thus included for analysis.

We then applied the method of qualitative thematic analysis [55] to the selected trip reports. The primary aim of this analysis was to provide a detailed account of one particular aspect of 5-MeO-DMT experiences—ego-dissolution—for the purposes of evaluating claims that self-consciousness can be either partially or totally lost during these experiences. Our analysis consisted of the following steps. The lead author first familiarized himself with the data. This involved holistically reading and re-reading the trip reports as narrative wholes describing a temporally evolving experience constituted by interrelated changes to consciousness. Then, the data were pre-processed by the lead author. This involved differentiating in the trip reports between direct descriptions of the acute experiences and any extraneous information, interpretation, explanation or judgement thereof that had been interpellated by the trip reporters. Reduced to their direct descriptions, trip reports in this form were then entered into NVivo v.14.23.3 [56] as the repository for our dataset. Using the coding functionality of NVivo, the lead author then manually coded for all described changes to consciousness in a bottom-up fashion and identified all descriptions that could be classified as involving ‘ego-dissolution’, i.e., which could refer to a disruption or loss of self-consciousness.

Applying Millière's [9] own theoretical framework that distinguishes between several different forms of self-consciousness, ‘ego-dissolution’ was subsequently sub-thematized in a top-down fashion into two categories: (i) descriptions identified as pertaining to a partial loss of self-consciousness in one or more forms, i.e. partial selflessness; and (ii) potential descriptions of all self-consciousness being lost, i.e., total selflessness. The category of partial selflessness was further sub-categorized according to the particular form of self-consciousness identified in the descriptions as having been disrupted or lost. These sub-categories (loss of cognitive self-consciousness, loss of mental ownership, loss of mental agency, loss of spatio-temporal self-location, loss of bodily ownership and loss of bodily agency) were defined in a top-down manner following Millière's categorizations, while we remained sensitive to new sub-categories arising from the data in a bottom-up manner during the coding process. Coding was then independently reviewed by the second author to confirm that it accurately reflected the descriptions at hand, with no disagreements arising in this regard. Key extracts were also agreed upon as especially lucid and detailed descriptions of ego-dissolution. Finally, we evaluated the claims of Millière and Letheby against the findings of our qualitative thematic analysis of the trip reports in order to determine whether 5-MeO-DMT can plausibly induce (i) partially selfless conscious states and/or (ii) totally selfless conscious states.

#### (ii) Results and discussion

Vaporised, insufflated or administered intravenously, 5-MeO-DMT typically induces—to a degree mediated by dose—a radical disintegration of basic structures of consciousness, thereby affecting the experience of space, time, embodiment, world and self (cf. [57,58]). A preliminary, naturalistic study of the phenomenology of 5-MeO-DMT experiences recently conducted by Timmermann *et al.* [58] refers to them as states of ‘deconstructed consciousness’ and further finds that 5-MeO-DMT, at its peak effects, can sometimes produce ‘a state of disconnection, loss of narrative and embodied aspects of the self and minimal phenomenal distinctions, while preserving wakefulness’ [58, pp.5–6]. This study does not, however, make any indication as to whether self-consciousness can be entirely lost—which is our primary interest here. Another recent study of the phenomenology of 5-MeO-DMT experiences conducted by Ermakova *et al.* [57] in a clinical setting found that at ‘high doses, a sense of self was generally retained, with only a few reporting complete dissolution of the sense of self’ [57, p.8]. However, no further specification was provided as to what exactly a ‘complete dissolution of the sense of self’ is supposed to entail.

Our analysis confirmed that descriptions of ego-dissolution are indeed common in 5-MeO-DMT reports, with 223 of the 411 trip reports (54.25%) including such descriptions. While Letheby's account of ego-dissolution is rather thin because he only addresses the possibility of *totally* selfless states, Millière's account is more sophisticated in also identifying different ways in which there can be *partially* selfless states. We now evaluate, against the findings of our analysis, the case for partial selflessness (as the disruption/loss of particular aspects or types of self-consciousness) and the case for total selflessness (as the loss of all self-consciousness). See table 1 below displays information concerning the frequency with which the reports analysed feature descriptions of specific types of change to self-consciousness, with these types defined in more detail in the subsequent text.

**Table 1.** Percentage of reports that feature descriptions of each categorised type of change to self-consciousness. Note that trip reports could feature descriptions that fulfil more than one category.

**type of change to self-consciousness**	**reports featuring change**
loss of cognitive self-consciousness	5.60%
loss of mental ownership or agency	2.19%
loss of spatio-temporal self-location	15.57%
loss of bodily ownership or agency	9.49%
enhancement or expansion of self-consciousness	12.90%
radically minimal self-consciousness	16.79%

*Partial selflessness*. Many forms and aspects of self-consciousness are affected by 5-MeO-DMT. In many cases, these changes correspond to those identified by Millière in his description of partial selflessness. However, our more comprehensive analysis also complements Millière's account by identifying types of transformation not discussed by him. We begin with the first form of self-consciousness identified by Millière: loss of cognitive self-consciousness. In his rendering, this involves an absence of self-reflective thoughts; of thinking of oneself as oneself [9, pp.7–9, 21]. He further specifies that cognitive self-consciousness is involved in ‘the planning and anticipation of future personal goals’ [9, p.8]. But further nuances can be added here. We also engage in self-reflective thoughts when we think of ourselves as someone with a complex personal identity: as having a life-story and certain identity-conferring values, relationships, interests, etc. This is an important detail, as self-reporters often describe losing all sense of personal identity (cf. [58, p.3]):

I [*sic*] had lost any sense of identity, of my humanity and of my surroundings […]. Everything enmeshed into a brutalizing cerebral tidal wave that obliterated any and all rational thought. My [*sic*] entire existence was condensed into a hysterical plunge into an incomprehensible abyss. I was not experiencing terror and dread—I was terror and dread [59].

Millière writes that 5-MeO-DMT intake can occasion losing the sense of mental ownership, such that one is no longer conscious of one's thoughts as one's own, and of mental agency, such that one loses the sense that one's mental actions are one's own [9, pp.14–18, 22–23]. We too identified reports expressing a loss of mental ownership and agency (cf. [57, p.9]). For example:

complete destruction of ego and loss of thought control. Within seconds I was overwhelmed by panic and anxiety. Negative thoughts raced through my head at an accelerating pace, but they weren't really ‘my thoughts.’ I had no conscious ability to analyse them [60].

We next confirm that spatial self-location can be lost during 5-MeO-DMT experiences ([9, pp.9, 21] cf. [58, p.3]). However, we also found descriptions of temporal self-location being confused or lost (featuring in 12.89% of reports); a dimension that Millière did not include. Temporal self-location can be confused or lost in several ways. These include (i) not being cognisant of ‘when’ one is relative to the past and future, (ii) time stopping or freezing, (iii) the sense of being outside of time or of ‘timelessness’ and (iv) losing all consciousness of time as such. Here is one example:

I slipped outside of time; for me, there was no such thing as time, because I was at the same time everything and nothing in the whole universe. I was an eternity, and I was a second [61].

We finally concur that the sense both of bodily ownership and of bodily agency can be lost, which Millière [9, p.5] respectively defines as ‘being conscious of one's body as one's own’ and ‘being conscious of one's bodily actions as one's own.’ Millière [9, pp.21–22] states that both forms of bodily self-consciousness can be lost by virtue of all awareness of the body being lost. This can produce the impression that one is a ‘disembodied’ mind or ‘pure consciousness’ (cf. [57, p.7]):

within 10 seconds I saw a mandala and could no longer feel my body…I no longer had a body. I was made entirely of thought [62].

But we also found descriptions of altered bodily awareness. While one is still aware of it, the body can be experienced (i) as a confusion of parts that no longer form a coherent body schema, (ii) as a depersonalized object, or (iii) as merged with the environment. Finally, (iv) bodily acts can also be experienced as involuntary. Below is an example of (i):

my perception of my body had been entirely scrambled; it was as if every nerve in my body had been randomly connected to the wrong input in my brain and all the inputs set off at once [63].

In his 2020 article, Millière distinguished six types of self-consciousness, and our analysis supports his claim that all six types can be lost during 5-MeO-DMT experiences. However, our more comprehensive study has added further nuance to his discussion of partial selflessness.

Before moving on to a consideration of total selflessness, it is worth pointing out that the scope of possible changes to self-consciousness that can occur in 5-MeO-DMT experiences is not limited to ego dissolution understood negatively in terms of a disruption or loss of self-consciousness. Although not identified by Millière or Letheby, we also found 53 trip reports (12.90%) that instead featured ‘ego enhancement’—i.e., experiencing features of one's self-consciousness more vividly, or ‘ego expansion’—i.e., experiencing new forms of self-consciousness. Here are a few examples:

— it felt like journeying to the centre of the onion. I felt like myself for the first time in I-don't-know-when. The kitchen, the hallway, the smell of the air all reminded me of my grandmother's house during my childhood [64].
— I lay down on the ground and was instantly immersed into a world that consisted solely of my ego. It was as though I was swimming in an ocean of myself. The intensity was beyond belief [65].

*Total selflessness*. Can the intake of 5-MeO-DMT also induce totally selfless conscious states? This concerns the key claim of both Letheby and Millière [8, pp.8, 15, 9, p.30]. In §2, we already presented a key argument against this claim. In philosophical discussions of self-consciousness, there is at least one central notion available that is markedly different from the six notions that Millière discusses and that concerns the ‘immanent reflexivity’ [31, p.162] of consciousness, i.e., the fact that a conscious mental state—in contrast to an unconscious mental state—is not merely outward-directed, but at the same time experientially manifest, i.e., given in and through and for itself. This is why Husserlians and neo-Brentanians, like Zahavi and Kriegel, for instance, have argued that self-consciousness is a necessary condition for phenomenal consciousness [40,41,66,67]. If this view is correct—and Letheby and Millière offer no principled arguments against it—it holds true *a priori* for all phenomenally conscious states and therefore also for all the states that are described in 5-MeO-DMT trip reports. Millière even concedes this point in a recent article, just as he accepts that all conscious mental states are characterized by for-me-ness, but then suggests that one should avoid classifying this fundamental form of self-consciousness as a form of self-consciousness [34, p.627]. At this point, the disagreement ceases being substantial, and Millière's main argument for the existence of totally selfless states turns out to rest on a terminological stipulation.

Let us in what follows raise a further worry concerning the empirical evidence discussed by Millière and Letheby. We have already mentioned that the key terms of reference—‘self,’ ‘ego,’ ‘I,’ ‘dissolution,’ etc.—are used imprecisely in ordinary language. In no case under study, bar one to be later addressed, did a 5-MeO-DMT self-reporter explicate what they meant by the terms they used when describing ego-dissolution. One can consequently neither assume that reporters have a clear and precise understanding of the terms they used, nor that their terms coincide with the way they are being used in the philosophical literature. A further reason for not taking descriptions of ego-dissolution too literally is given by self-reporters themselves. Many emphasise that 5-MeO-DMT produced such rapid and bizarre changes to their self-consciousness that they cannot accurately describe, comprehend or even remember what occurred (cf. [57, p.9, 58, p.4]). Pollan, for instance, states concerning his experience of ego-dissolution that ‘words fail […] I'm grasping at metaphor in the hope of forming some stable and shareable concept of what was unfolding in my mind’ [21, p.277]. Both Letheby and Millière exclude this detail when abridging Pollan's report. Consider a further example:

I'm confused as hell, and hardly know how to describe it—tagging any words to its shining essence feels hideously futile […]. Ego loss, for ‘me’ (whatever that is), is something that I ‘myself’ can't really make sense of after the phenomenon is over. I only remember it as a pale fact, not a reality [68].

Of course, the existence of many 5-MeO-DMT trip reports means that the experience is not entirely indescribable. But the fact that many self-reporters emphasize the shortcomings of their descriptions urges further caution against simply taking them literally and at face value.

A central point to be considered next concerns the affective dimension of the experience (cf. [57, p.8, 58, p.3]). Self-reporters commonly describe their experience of ego-dissolution as involving profound feelings of confusion, terror and panic or even ‘infinite bliss, joy and ecstasy,’ as Letheby [8, p.11] himself admits. As Pollan describes, for example:

the terror didn't disappear with the extinction of my ‘I.’ Whatever allowed me to register this experience, the post-egoic awareness I'd first experienced on mushrooms, was now consumed in the flames of terror too. In fact, every touchstone that tells us ‘I exist’ was annihilated, and yet I remained conscious. ‘Is this what death feels like? Could this be it?’ That was the thought, though there was no longer a thinker to have it [21, p.277].

It is difficult to understand how the self-reporters could have emotional responses to the experience of ego-dissolution unless the experience mattered to *someone*. Forde similarly states that ‘if Pollan was seized by terror during the experience then *who* was terrified? If his well-being was perceived to be under threat then does this not highlight some sense of self-preservation?’ [2, p.51]. In Pollan's case, the statement following his morbid musing, namely, ‘that was the thought, though there was no longer a thinker to have it’ may well refer to an absence of the features that constitute Pollan's ordinary sense of self or ‘personal identity,’ i.e., his interests, values, desires, along with a sense of agency and ownership over mind and body, and so on. The plausibility of this interpretation stems from Pollan's own report, where he provides an explanation of what he means by ‘ego’ when reflecting on his 5-MeO-DMT experience, although Letheby and Millière fail to include it. Pollan describes the ‘ego’ as follows:

there is much more to consciousness than the ego […]. But the ego, that inner neurotic who insists on running the mental show. That stingy, vigilant security guard admits only the narrowest bandwidth of reality […]. It's really good at performing all those activities that natural selection values: getting ahead, getting liked and loved, getting fed, getting laid. Keeping us on task, it is a ferocious editor of anything that might distract us from the work at hand, whether that means regulating our access to memories and strong emotions from within or news of the world without [21, pp.288–289].

As should be clear from a statement like this, what Pollan means by ‘ego’ is very different from the minimal sense of self captured by the term ‘for-me-ness,’ which, in the sense we are applying it, refers to the subjective and perspectival character of consciousness (see [41]). To conclude that Pollan is describing the obliteration of the latter when talking of ‘the extinction of my ‘I’’ would be unwarranted. This, however, is precisely Letheby's claim, who is adamant that reports such as Pollan's can be grasped in their ‘utter uniqueness only by appreciating that (they lack) what remains constant across nearly all conscious experiences, including those of inserted thoughts: the apparent fact (the seeming) that experiential objects are ‘given to (some)one in a distinctly first-personal way,’’ i.e., for-me-ness [8, p.18].^8^ Let us instead propose what we take to be a more plausible interpretation. The ‘extreme ego-dissolution’ that is reported in 69 trip reports (16.79%) involving 5-MeO-DMT is indeed extraordinary, and it is so by virtue of entailing a *radically minimal* form of self-consciousness.

Here, ‘radically minimal’ means that the self-consciousness is stripped of *nearly* all features except for-me-ness. In the absence of any further identifying features or properties—by virtue of the loss of all other forms of self-consciousness—the ‘me’ of for-me-ness is radically underdetermined; it is reduced to a ‘bare subjectivity’ [2, p.58]. In the absence of further information regarding name, gender, physical appearance, profession, goals, memories, etc., no further identifying properties are attributable to oneself, and the nature of ‘who’ one is as the one who is self-conscious is underdetermined. ‘You’ are nowhere, at no time, nameless, disembodied and lacking control of all thoughts and movements. ‘You’ are just (barely) self-conscious.

On this interpretation, one can understand why some self-reporters experience profound feelings of terror and confusion in a state of extreme ego-dissolution and even have thoughts or fears about whether they are dead. As one self-reporter writes, ‘I felt that there was nothing to me’ [69]. This is a state that is profoundly difficult to comprehend, describe, accept and cope with, but it would be wrong to deny that there is a first-person way in which extreme ego-dissolution is experienced. Consider, by comparison, the terror one might feel if one woke up in a state of complete amnesia and found oneself immobilized in a dark room (cf. [40]). Moreover, the experience can differ from subject to subject, notably with respect to whether it is experienced as terrifying, joyful, bewildering, etc., but perhaps also with respect to the process by which radically minimal self-consciousness is reached (i.e., the sequence by which the six other forms of self-consciousness are lost), the duration of the state, and the process by which ordinary self-consciousness is regained (sometimes being experienced as a euphoric rebirth, as described in 7.54% of the reports; cf. [57, p.9]).

A possible riposte can now be considered. Millière states that ‘a number of reports of putative selfless states precisely avoid using the first-person pronoun, or explicitly point out its inadequacy’ [9, p.32]. He argues that this provides strong reason to think that a totally selfless state was experienced, i.e., there was thereby no ‘self’ to which to refer. However, an alternative interpretation is available. The difficulty or avoidance of using first-person pronouns when describing ego-dissolution might reflect a profound perplexity on the part of the self-reporters (cf. [50, p.22]). They are still self-aware in the sense of having radically minimal self-consciousness, but, in these extreme states, they cannot figure out ‘who’ they are. Absent awareness of any forms and features of self-consciousness other than the bare consciousness of one's existence, the ‘me’ of for-me-ness during extreme ego-dissolution loses all its usual familiarity.^9^ As one self-reporter states, ‘I most certainly did not know what ‘me’ was’ [73]. Consider also this telling example:

I (?) attempt to get a grip on myself […]. I have absolutely no control over this experience or my(?)self. Again I attempt to express unease verbally, and I am unsure of my success, but again I receive reassurance from the part of my(?)self that is sitter no. 2. I can't hear or understand her words, but I know that she/I(?) is calm and confident, and that helps me to stay on the side of the line that is fright and thrill, and not on the other side, on which lies panic and terror and madness […]. Then there are hints of the world returning. I(!) reach out with my(!) hands and pull sitter no. 2 to my body, pressing my(!) face into her neck, willing myself to slide my soul into her body, since I know she is in the world, where I would like to be again [74].

When reporting extreme ego-dissolution after the fact, it can be difficult to retrospectively refer to oneself in that state as ‘I’ or ‘me.’ The radically minimal self-consciousness experienced bears so little resemblance to one's ordinary self-consciousness that it is almost as if one is dealing with two different subjects. This helps to clear up apparent contradictions in some descriptions of extreme ego-dissolution, such as when self-reporters state that they had, on the one hand, no sense of self but, on the other hand, express a transformed sense of self and use pronouns to refer to themselves as the subject who experienced extreme ego-dissolution. For example, ‘the ‘I’ that emerges is not the one who went in’ [75], ‘I became eternity, except that I was no longer me’ [76], ‘I was not me, I was nothing but undefinable fear’ [77], or ‘I lost my mind, my soul, my ego and now I have to find where I left it’ [78]. However, we stress that it is only ‘almost like’ being two subjects. Even in cases of extreme ego-dissolution, some form of for-me-ness is retained.

Let us finally note some broader implications of our qualitative analysis. First, our analysis has contributed to philosophical discussions of self-consciousness by pointing to the existence of a pure manifestation of minimal self-consciousness, namely, ‘radically minimal self-consciousness.’ It is ‘pure’ in the sense that minimal self-consciousness alone is present in extreme ego-dissolution while all other types of self-consciousness are absent, whereas in ordinary experience minimal self-consciousness rarely occurs on its own. This finding demonstrates that the qualitative analysis of drug experiences, and extreme forms of experience more broadly, can offer valuable input to philosophical work on the nature of self-consciousness. Second, this notion of radically minimal self-consciousness may prove helpful for understanding alleged cases of ‘selflessness’ in other forms of anomalous experience, such as in meditative experiences [22], near-death experiences [79], and thought-insertion in schizophrenia [70]. The idea that thought-insertion can involve a total absence of for-me-ness, i.e., total selflessness, has already been rebutted [70]. What may appear to be instances of total selflessness in other cases may instead be conceived as instances of pure minimal self-consciousness. Finally, our analysis demonstrates that the most reliable way to study ego-dissolution in general—however it may come about—is arguably by way of a theoretically *and* qualitatively well-grounded analysis of first-person reports such that vaguely construed empirical findings and abstract speculations are avoided.

#### (iii) Limitations

As we only analysed anonymous and/or already published trip reports, our study is limited in the following two respects. First, ambiguities in descriptions of ego-dissolution could not be further discussed or clarified with self-reporters. Semi-structured interviews are thus needed to elicit clearer and more detailed descriptions from subjects in order to more reliably differentiate direct descriptions of the acute experience and potential retrospective biases, as well as to explicate or revise the initial account of 5-MeO-DMT-induced ego-dissolution presented in this paper. Second, we cannot assume that the anonymous trip reports are reliable with respect to accurate dosage reporting, and they also provide little information concerning key contextual factors (personality, gender, biography, etc.) and setting (surrounding physical and social environment). No conclusions can thus be drawn regarding how dosage and contextual factors mediate the occurrence and experience of ego-dissolution. But since we have focused on ego-dissolution occurring in 5-MeO-DMT experiences by whatever dose and under whatever circumstances, the unsettled nature of these issues has no implications for our analysis. What's more, 5-MeO-DMT is a rarely used substance in naturalistic and clinical settings alike. As such, 5-MeO-DMT trip reports are rare in publication. Given that Erowid.org archives the majority of extant 5-MeO-DMT trip reports, it made sense to begin analysis with this dataset, despite the already mentioned limitations of analysing anonymous trip reports. Considering these limitations, we present our analysis as exploratory and our conclusions as open to explication or revision.

A final limitation is that our qualitative analysis included only trip reports involving 5-MeO-DMT, given that they have been presented as the clearest evidence for totally selfless states of consciousness. Qualitative research on reports of ego-dissolution induced by other psychedelics should be compared in future research with reports of ego-dissolution induced by 5-MeO-DMT in order to develop a more comprehensive understanding of the general phenomenon. Such comparative work is beyond the scope of this paper. However, we note that a recent phenomenological qualitative study of 1895 trip reports by one of us found that classic psychedelics (LSD, psilocybin, N,N-DMT and mescaline) commonly induce radical transformations of self-consciousness, and that this can include the loss of all types *except* for minimal self-consciousness [80, pp. 235–243, 456–459].

## 5. Conclusion

In this paper, we have critiqued recent claims concerning ego-dissolution induced by 5-MeO-DMT. We first argued that neither Letheby nor Millière are operating with truly minimal notions of self and self-consciousness and that their claim that ego-dissolution can take the form of total selflessness is consequently either not warranted or simply based on a terminological stipulation. We subsequently focused upon the empirical validity of the claims that 5-MeO-DMT trip reports provide clear evidence of both partial and total selflessness. We initially raised concerns about the methodological underdetermination of Letheby's and Millière's use of such trip reports. We then confirmed, on the basis of our own extensive qualitative analysis of 5-MeO-DMT trip reports, that ego-dissolution can involve partial selflessness. We further argued that there are principled reasons for contesting that extreme ego-dissolution in 5-MeO-DMT experiences amounts to total selflessness, and that the very idea that it is possible to find empirical exceptions to the claim that consciousness is characterized by self-consciousness is misguided. Extreme ego-dissolution is instead extraordinary, we proposed, by virtue of exemplifying a *radically minimal*, i.e., pure, form of self-consciousness. For-me-ness is maintained, but without any further specifying aspects and forms of self-consciousness, the ‘me,’ i.e., self-identity of the subject, is experienced as unfamiliar and underdetermined. We thus insisted that there is something it is like to first-personally experience extreme ego-dissolution.

One reason why the study of psychedelic ego-dissolution is theoretically rewarding is that it allows for a better understanding of the dependency relations that hold (or do not hold) between different forms of self-consciousness. In ordinary life, different forms of self-consciousness often co-occur, but thinking about exceptional cases ‘is indispensable if we wish to avoid mistaking accidental regularities for regularities which reflect a deeper truth about the world’ [81, p.463]. As our critiques should have demonstrated, however, more conceptually sophisticated and empirically rigorous studies of psychedelic ego-dissolution are needed if we are to clearly and reliably determine its experiential nature, identify its potential psychotherapeutic benefits and neural correlates, as well as harvest whatever insights into the fundamental nature of consciousness that its study might offer.

## Data Availability

The 412 5-MeO-DMT trip reports analysed from Erowid.org are publicly and freely accessible on Erowid.org via publication [82].

## Footnotes

^1^
Letheby and Gerrans employ the terms ‘self-awareness’ and ‘self-consciousness’ interchangeably in different publications. For the sake of simplicity, we will in the following stick to the term ‘self-consciousness.’

^2^
In her article, Guillot acknowledges that Zahavi's prior work on for-me-ness also contains a different rendering of the term, namely, one according to which for-me-ness should not be understood as a relation between two distinct particulars, the subject and the experience, but where the property of being ‘for me’ is rather constituted by the (inner) awareness that an ongoing experience has of itself [20, p.29]. This rendering is, however, overlooked by Letheby, which is somewhat unfortunate, since one of Letheby's aims is to criticize Zahavi's account.

^3^
When Millière writes that the term self-consciousness “naturally refers to the subject's consciousness *of herself* – just as ‘time consciousness’ and ‘bodily consciousness’ typically refer to the subject's consciousness *of time* and *of her body* respectively” [9, p.29], we would also have to disagree. The classical work on time consciousness, Husserl's *Lectures on the Phenomenology of the Consciousness of Internal Time* is not primarily an analysis of the consciousness *of* time, i.e., an analysis of those experiences that have time as their intentional object, but is rather an analysis of the way in which the intrinsic temporalization of the stream of consciousness affects and enables a variety of intentional accomplishments, including object perception and recollection [35].

^4^
For an explicit discussion of these misunderstandings, see [37].

^5^
On some accounts, it makes little sense to claim that an experience is given in a *distinct* manner to the subject of the experience, since it is never given in any other way. The only experiences I am acquainted with are my own and whatever experiences others might have are never given to me at all. As influential as this view has been, it has been subjected to criticism by both classical phenomenologists such as Scheler [43, pp. 220, 260] and by recent contributors to the social cognition debate (e.g., [44–46], who, by appealing to notions such as social perception or person perception, have argued that we can in fact see the other's elation or doubt in his or her face, hear the other's trepidation, impatience or bewilderment in her voice, feel the other's enthusiasm in his handshake, grasp his mood in his posture, and see her determination and persistence in her actions. For an extensive discussion of the relation between self-experience and interpersonal understanding see [41] and [47].

^6^

https://erowid.org/experiences/subs/exp_5MeODMT.shtml.

^7^
Trip reports will heretofore be cited according to their pseudonymous author identification as listed on Erowid.org.

^8^
Letheby's reference to ‘experiential objects’ is incidentally somewhat confusing. On the account that he seeks to criticize it is not the objects of experience (i.e., the vase, the tree, the sunset) that are given first-personally, but the acts of experience.

^9^
Compare also the case of thought-insertion in schizophrenia, where for-me-ness has been described as losing its usual familiarity, with the consequence that reflective self-ascription becomes difficult [70–72].

## Theme

One contribution of 17 to a discussion meeting issue ‘From being to identity: analysis and synthesis of the self’.
